# Models of Hepatocellular Carcinoma and Biomarker Strategy

**DOI:** 10.3390/cancers2031441

**Published:** 2010-07-07

**Authors:** Cedo M. Bagi, Catharine J. Andresen

**Affiliations:** Global Science & Technology, PGRD, Pfizer Inc, Groton, CT 06340, USA; E-Mail: catharine.j.andresen@pfizer.com

**Keywords:** HCC, cell cocktail, orthotopic models, liver vasculature, CEUS, PET, MRI, histology

## Abstract

The overwhelming need to improve preclinical models in oncology has stimulated research efforts to refine and validate robust orthotopic models that closely mimic the disease population and therefore have the potential to better predict clinical outcome with novel therapies. Sophisticated technologies including bioluminescence, contrast enhanced ultrasound imaging, positron emission tomography, computed tomography and magnetic resonance imaging have been added to existing serum- and histology-based biomarkers to assist with patient selection and the design of clinical trials. The rationale for the use of human hepatocellular carcinoma (HCC) cell lines, implementation of xenograft and orthotopic animal models and utilization of available biomarkers have been discussed, providing guidelines to facilitate preclinical research for the development of treatments for HCC patients.

## 1. Introduction

Hepatocellular carcinoma (HCC) is the fifth most common cancer worldwide and its incidence in the United States and other countries has been steadily increasing over the past 25 years [1,2]. Given the large number of HCC patients, there is a strong mandate to develop relevant animal models and biomarkers [3] that will enable accurate translation from preclinical research to clinical practice when developing new single-agent or combination treatments for HCC. Results from several studies conducted in our laboratory were used to illustrate challenges and provide guidance to scientists working on preclinical models of HCC. Technical hurdles and alternative approaches deployed when testing the efficacy and safety of anticancer compounds in animal models of HCC were also discussed, as well as the prognostic and translational value of several biomarkers that are most frequently used for efficacy assessment of novel therapies in clinical studies.

Advanced HCC is highly refractive to currently available chemotherapies, and patients suffering from HCC often develop drug resistance during treatment. Several chemotherapeutic agents have been tested in patients with advanced HCC; however, the results were disappointing and life-threatening side effects frequently limit prolonged treatment [4]. One of the chemotherapeutics tested in HCC patients, doxorubicin (Adriamycin^®^, Adria Laboratories), showed rather low efficacy; however, with a response rate of 10-15%, doxorubicin remains the most effective single agent currently available [5]. Recently, a VEGF inhibitor, sorafenib (Nexavar^®^, Bayer), significantly prolonged the time to tumor progression, increased overall survival in a placebo-controlled phase III study in HCC patients [6,7] and significantly increased the effectiveness of doxorubicin treatment in those suffering from advanced HCC [8]. Because liver tumors are known to be highly vascular, another oral tyrosine kinase inhibitor sunitinib (Sutent^®^, Pfizer Inc.) was also tested in phase II clinical trials in HCC patients showing that HCC could be susceptible to treatment with anti-angiogenic compounds [9,10].

Preclinical experimentation allows for simultaneous longitudinal implementation of various technologies and biomarkers to monitor the tumor take rate, growth and response to treatment as well as to confirm and correlate histological and histochemical results at various time points with serum or imaging biomarkers, which cannot be determined in HCC patients. Different animal models and biomarkers, such as serum alpha-fetoprotein (AFP), bioluminescence imaging (BLI), contrast enhanced ultrasound imaging (CEUS), positron emission tomography (PET), computed tomography (CT) and immunohistochemistry (IHC), can be utilized to build confidence in the efficacy and safety of tested compounds during preclinical research and help to develop biomarker strategies and design clinical trials.

## 2. Discussion

### 2.1. *In Vitro* Studies

#### HCC Cell Lines and *in Vitro* Efficacy

Similar to other cancers, *in vitro* testing of human HCC cell lines is usually an early step in the process of anticancer drug discovery that involves evaluating viability, cell proliferation, clonogenicity and apoptosis. *In vitro* results from our studies in four commonly used human HCC cell lines: Huh7.5, HepG2, Hep3B and SK-Hep1, showed that sunitinib alone moderately inhibits proliferation in all four HCC cell lines with IC_50_s in the low micromolar or high nanomolar range. Doxorubicin used as a monotherapy was also effective; the IC_50_ was in the low nanomolar range in all four HCC cell lines deployed. Combining doxorubicin and sunitinib resulted in additive inhibitory effects on cell proliferation in all cell lines used in the study; however, none of the cell lines were very sensitive or resistant to imposed treatments [11]. Mechanistically, the increased *in vitro* therapeutic efficacy with the drug combination was mainly due to the induction of apoptosis and inhibition of proliferation.

#### 2.2. *In Vivo* Studies

##### 2.2.1. Animal Models of HCC—General Remarks

**Figure 1:**
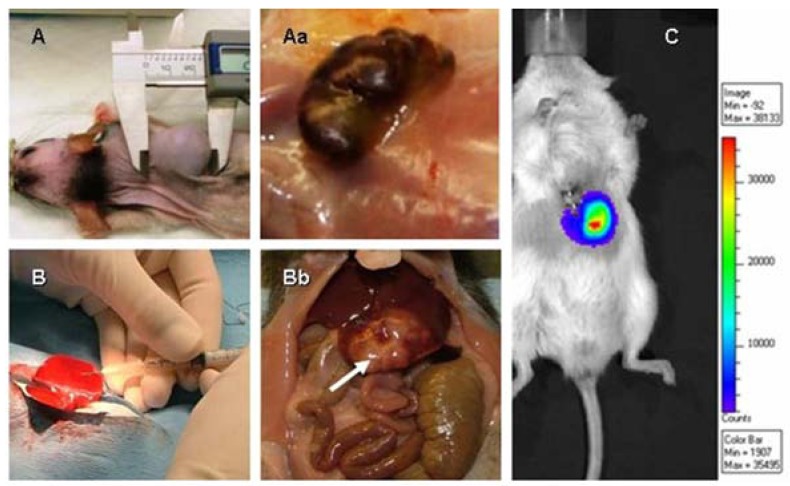
Xenografts of HCC tumors in nude rats measured by caliper (**A**) and the appearance of a highly vascularized subcutaneous tumor (**Aa**). Inoculation of HCC cells into the livers of nude rats to create the orthotopic model (**B**). Inoculation of HCC cells into the liver results in restriction of the tumor mass to the liver lobe in which the tumor cells were injected (**Bb**, arrow indicates tumor mass). Bioluminescent imaging of the orthotopic tumor (**C**) using IVIS technology is a helpful method to confirm the tumor take rate, monitor tumor progression and assess therapeutic outcome.

While results obtained using cell cultures provide important information regarding drug efficacy and mechanisms of action, *in vitro* systems lack the power to recapitulate the complex relationship between the tumor and its microenvironment, including local blood supply and angiogenesis, interactions between tumor cells and the organ where the tumor resides, and the influence of hormones, growth factors and cytokines on tumor growth and survival. Scientists usually start *in vivo* oncology work with subcutaneous (SC) xenograft models because these are relatively easy to perform; tumors are externally placed and simple caliper measurement of tumor size provides insight regarding compound efficacy measured as inhibition of tumor growth (TGI) [Figure 1A and 1Aa]. The main goal of studies using xenograft models is to confirm that the “targeted” therapy under investigation hits the intended target that should be present in the tumor cell line used in the study. On the other hand, orthotopic models of liver tumors are labor-intensive as they require surgical inoculation of tumor cells into the liver (or spleen) and the use of sophisticated imaging technologies and serum biomarkers to monitor the tumor take rate, tumor growth and effects of therapy on tumor progression. However, because in orthotopic models the tumor is placed in its native environment (organ), studies performed in these models provide data with higher value to clinicians, including the exposure of the tumor to drug at the organ level, rate of tumor growth in its natural milieu and finally preclinical evaluation of biomarkers that are available for particular tumor types. Obtained results from studies in orthotopic models should lead to the development of treatment and biomarker strategies in clinical trials (Figure 1B, 1Bb) [12,13,14,15]. Therefore, xenograft and orthotopic models are complementary, and both models have a place in the screening strategy of novel therapies.

##### 2.2.2. Use of Cell Cocktails to Better Predict Clinical Outcome

Selecting the cell line(s) for an animal study is often challenging because the scientist must decide which cell line, from all cell lines available for a particular tumor type, best represents a particular human cancer, and selecting only one cell line that responds to exact treatment could be very misleading when it comes to clinical outcome [16]. Even if only one tumor cell line is selected for an *in vivo* study, testing combination therapy using two or three drugs adds to the complexity of the study design and study execution. For example, if the study design calls for the use of only one imaging method and one biomarker, then each methodology must be deployed in the same model/study in all study groups treated with sole therapy as well as in study group(s) treated with two or three drugs used in combination. The practicality of study conduct end execution becomes even more complex if the study design requires testing a combination of two drugs in several HCC cell lines. Such a study will require 16 different study groups and with a minimum of eight animals per group to achieve statistical significance, anticipated study conduct will become very challenging for any lab even if a simple subcutaneous xenograft model is deployed. Because the number of available tumor cell lines is constantly increasing, testing novel compounds in all available cell lines becomes increasingly difficult, in particular since the research community in general lacks firm criteria to guide the decision on which HCC cell line has relevance for a particular patient population. In addition, tumor cells grown in culture are susceptible to undergoing genetic alterations not found in the original tumor, such that xenografts created by these cells may differ from those of naturally occurring tumors [17]. 

To improve the robustness and predictive value of *in vivo* data, we propose the use of cell cocktails composed of several HCC cell lines to create xenograft or orthotopic models as the next step in screening cascades aimed at building confidence in compound efficacy in *in vivo* studies in single-cell xenografts have confirmed therapeutic effect. Also, the deployment of cell cocktails allows for the assessment of eventual resistance to treatment of individual cell line(s) used to make the cocktail, pending same exposure to drug(s) and treatment duration. Finally, the use of tumor cell cocktails if and when possible will reduce the amount of drug needed for testing, lessen the time and resources needed for preclinical studies without compromising data quality and integrity as well as support the 3Rs animal welfare paradigm (Replace, Refine and Reduce) [18]. When using proposed cell cocktail alternatives, scientists should indicate the rationale for including or excluding particular cell line(s) from their cocktail and ensure that they have a follow-up strategy in place if they attempt to harvest the tumor tissue from the *in vivo* study to assess which cell line(s) used in the cell cocktail did not respond to treatment. 

Alternatively, human biopsies instead of tumor cell lines can be used to create xenograft or orthotopic models, but caveats to this approach are the high cost of procedures and transfer of biopsies along with safety issues due to a high rate of hepatitis C (HCV) and hepatitis B (HBV) infections in HCC patients [19,20,21,22]. In addition, the use of biomarkers such as AFP or IVIS imaging in studies using human biopsies will not always be possible.

##### 2.2.3. Liver Vasculature—Relevance for Models of HCC

**Figure 2:**
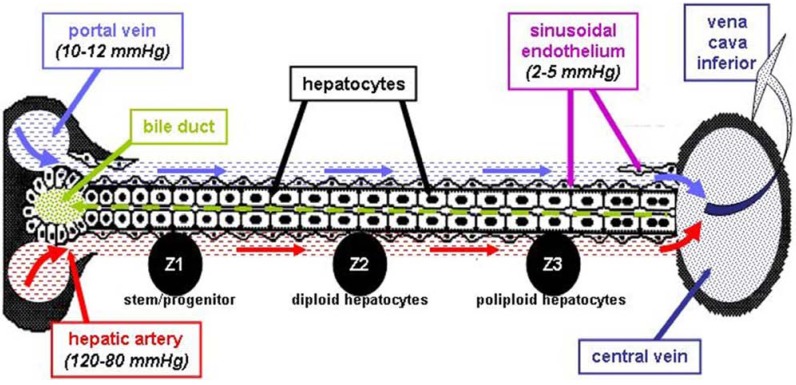
A schematic representation of the nutritional (hepatic artery) and functional (portal vein) blood supply in the liver, emphasizing differences in blood pressure between the two.

The local vasculature of the liver is extremely complex and is composed of nutritional and functional blood supplies that in addition to neo-vasculature created by tumor itself, collectively support tumor growth within the liver. In brief, total liver blood flow represents approximately 25% of the cardiac output, with roughly 100-130 mL of blood circulating through 100 g of liver tissue per minute, and this number is fairly constant across all mammalian species. The hepatic artery accounts for about 65% of the oxygen supply to the liver [23,24,25]. The initial difference in blood pressure (BP) between the hepatic artery (80–120 mmHg) and portal vein (10–12 mmHg) tends to decrease deep in the liver parenchyma, where BP is only about 2-5 mmHg in sinusoidal endothelium (Figure 2). Slow circulation in the liver and an intense vascular network allows the tumor to establish, survive and eventually metastasize [26]. Unlike the dual vascular supply of the normal hepatic parenchyma that is provided by vessels arising from both the systemic arterial circulation and the portal venous circulation, the blood flow to the tumor is carried almost entirely by systemic arterial vessels [27,28]. The portal vein is largely responsible for functional vasculature and proper functioning of the liver; however, oxygen and nutrient supply through the portal vein is respectable, and outside edges of the tumor use this alternate route to survive and spread to surrounding tissues [29]. In addition, local vascularization in the liver of cancer patients can be further complicated by liver cirrhosis, an irreversible fibrous scarring associated with hepatocellular regeneration that is characterized by diffuse disorganization of the normal hepatic structure of regenerative nodules and fibrotic tissue [30,31]. Based on the complex vascular events and microenvironment of the liver that plays a role in tumor growth and spreading, only orthotopic liver tumor models can provide the level of complexity that is needed to reliably evaluate the antitumor effects of compounds under investigation in preclinical studies. Consequently, establishing drug efficacy in subcutaneous tumors does not guarantee the translation of efficacy in orthotopic preclinical models to patients treated for HCC in the clinic.

##### 2.2.4. Drug Delivery—Clinical and Preclinical Relevance

**Figure 3:**
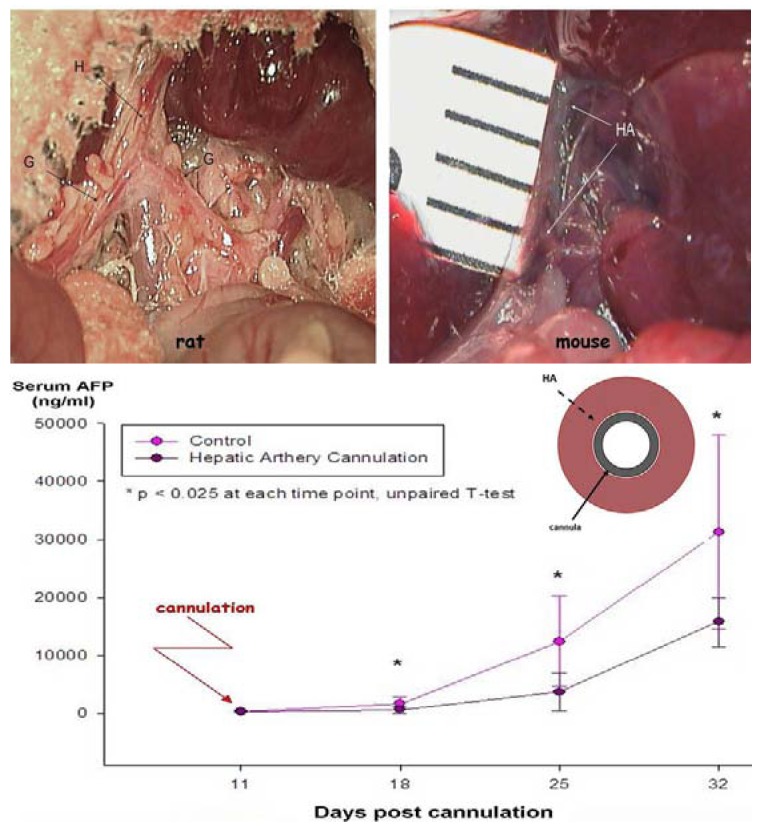
The hepatic and gastric arteries in the rat (upper left panel) and mouse (upper right panel) and the effect that prolonged cannulation has on growth of an intrahepatic tumor assessed by alpha-fetoprotein (lower panel). Prolonged cannulation of the hepatic artery results in reduced arterial diameter, leading to a reduction in oxygen supply to the tumor. H and HA—hepatic artery, G—gastro-duodenal artery.

**Figure 4:**
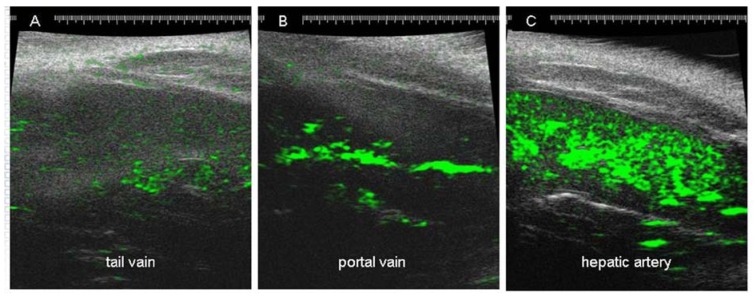
Ultrasound images of rat livers following bolus injection of microbubbles (50 mL) into the tail vein (**A**) portal vein (**B**) or hepatic artery (**C**). Take of the microbubble contrast (green) by the liver is very high if the injection is made through hepatic artery or portal vein, but poor if injection is made through the tail vein.

One of the most clinically effective ways to deliver drugs to the liver is through the hepatic artery because it allows continuous infusion directly into the arterial bed from which the tumor derives nearly all of its blood supply [32,33]. Injection of HCC cells into the livers of immunodeficient rodents causes progressive growth of tumor cells in the liver [34,35,36]. The use of orthotopic animal models allows for cannulation of the hepatic artery and portal vein to deliver drugs directly to the liver, thus mimicking the clinical arrangement. Because the hepatic artery in rats and mice is fairly small, prolonged catheterization of the artery will impact tumor growth and that should be taken into account when interpreting results obtained in cannulated animals (Figure 3). However, a single bolus injection of a drug or contrast agent through the hepatic artery does not impact tumor growth in the liver and can be safely used for drug delivery. Due to technical difficulties associated with intra-arterial drug delivery, the most common way of dosing animals in preclinical studies is via tail-vein injections. Even though tail-vein injections have little similarity with the clinical setup, this method is acceptable as long as the frequency of repeated dosing is manageable (danger of local tissue damage and necrosis) and a serum level of drug has been confirmed to ensure proper delivery. The differences in the microbubble concentrations in the liver following tail-vein, portal vein and hepatic artery delivery are shown in Figure 4. 

##### 2.2.5. Use of Biomarkers in Preclinical Models of HCC

Clinical diagnosis and monitoring of HCC in patients consists of a blood test for elevated AFP concentrations followed by structural imaging utilizing one of several imaging modalities that are currently available. The most frequently used imaging methods are CEUS, contrast CT and MRI. Also, PET imaging with FDG and/or FLT is increasingly being used both preclinically and in clinical practice [37,38,39,40,41,42]. All of the imaging technologies mentioned above are validated for preclinical and clinical use, therefore providing a valuable translational tool to assess the efficacy of novel therapies in animal models of HCC as well as in the clinic. In addition, technological advances including the availability of safe contrast agents continue to add value to current methods. Finally, novel imaging technologies such as BLI are useful tools in the preclinical setup but with limited translational value for clinical studies [43,44].

AFP is the primary serum biomarker for HCC and is elevated in up to 40% of patients with early-stage HCC, with expression increasing in advanced disease [45,46]. Although AFP is not specific for HCC and may be seen clinically in nonmalignant conditions such as chronic hepatitis, cirrhosis or fulminate hepatic failure, the elevation of AFP and confirming ultrasound findings clearly indicate malignant disease. AFP in preclinical studies is a reliable biomarker of the tumor take rate and growth. Serum AFP correlates well with the measurement of tumor size by caliper, weight of excised tumor tissue and ultrasound measurement of tumor volume. AFP and HFUS are valuable tools in preclinical studies for assessing therapy in orthotopic models and provide excellent translation to the clinic [47].

US technology using microbubbles as a contrast agent is known to be an extremely useful tool for non-invasive assessment of liver vascular structure [48]. CEUS is being used in both preclinical and clinical studies to diagnose liver tumors and/or liver metastases, to monitor disease progression and to assess the efficacy of antitumor therapies. Besides its diagnostic value, US technology has the potential to be deployed in the clinic as a treatment that supports the use of technology by using targeted destruction of drug-loaded microbubbles at the tumor site to expose the tumor to a high drug concentration and reduce the risk of systemic toxicity [49,50,51]. The combination of AFP and CEUS provides a powerful tool to assess therapeutic effects on intrahepatic tumors, and well-designed animal studies should also include tumor histology to cross-validate CEUS data with histology, something that is hard to achieve in the clinical setup. 

BLI is based on the detection of light emitted by living cells expressing a luciferase gene [43,44]. Stable transfection of luciferase in HCC cells and inoculation of labeled tumor cells into the liver allows for noninvasive monitoring of the tumor take rate, tumor progression and treatment effectiveness, thus optimizing preclinical study protocols. BLI provides an additional tool for monitoring orthotopic tumor models where the tumor is not visible and thus the estimate of tumor size is dependent on the use of a serum biomarker such as AFP. While AFP has been shown to correlate nicely with tumor size, it does not provide the location of tumor cells, thus in models of HCC tumor metastasis, tools such as IVIS are extremely valuable in locating the tumor.

The use of PET and MRI imaging is increasing as these technologies are becoming more affordable. However, due to the high cost, associated radiation, high level of skills required for data acquisition and interpretation as well as need for other labor-intensive procedures, PET and MRI imaging should be reserved only for advanced projects with a specific question in mind that cannot be answered using other imaging modalities. 

## 3. Conclusions

There are multiple and independent biomarkers, including serum AFP, CEUS, PET and MRI imaging and tissue IHC, available for use in preclinical animal studies investigating novel compounds and therapeutic combination paradigms for HCC. The use of the cell cocktail approach, orthotopic models and careful study design as well as selection of biomarkers can maximize the value of *in vivo* research and ensure translation of results to the clinical situation. A thorough preclinical approach to HCC research should build confidence in compound efficacy as well as safety and lead to the discovery of novel compounds or more efficacious drug combinations for treating HCC.

## Declare

Part of this manuscript was presented by Cedo Bagi as oral presentation at 7th Annual Cancer Drugs, Research & Development Conference, San Diego, CA, January 28–30, 2010.
